# Identification of Novel Simulants for Toxic Industrial Chemicals and Chemical Warfare Agents for Human Decontamination Studies: A Systematic Review and Categorisation of Physicochemical Characteristics

**DOI:** 10.3390/ijerph18168681

**Published:** 2021-08-17

**Authors:** Thomas James, Samuel Collins, Tim Marczylo

**Affiliations:** Centre for Radiation, Chemicals and Environmental Hazards (CRCE), Public Health England, Chilton OX11 0RQ, UK; samuel.collins@phe.gov.uk (S.C.); tim.marczylo@phe.gov.uk (T.M.)

**Keywords:** human volunteer, mass decontamination, simulants, CWA, TIC

## Abstract

Chemical simulants have long been used in human trials of mass decontamination to determine the efficacy of decontamination interventions against more toxic agents. Until now, reliance has mostly been on individual chemicals as surrogates to specific agents (e.g., methyl salicylate for sulphur mustard). A literature review was conducted to identify chemicals that had been previously tested on human volunteers and that represent diverse physicochemical characteristics in order to create a repository for chemical simulants. Of the 171 unique chemicals identified, 78 were discounted for the risk they could pose to human volunteers, 39 were deemed suitable for use, and a further 54 were considered to be possible simulants but would require further research. Suitable simulants included both solid and liquid chemicals spanning a wide range of physicochemical properties including molecular weight, octanol/water partition coefficient, vapour pressure, and solubility. This review identifies an array of potential simulants suitable for use in human volunteer decontamination studies and is of relevance to future studies on systemic absorption and surface decontamination.

## 1. Introduction

Chemical, Biological, Radiological, and Nuclear (CBRN) incidents, whether accidental or intentional, often require some form of casualty decontamination, which in the UK, can include the following: dry or wet improvised decontamination during an Initial Operational Response (IOR); interim decontamination such as a ladder-pipe system using more structured front-line appliances; or mass decontamination during the Specialist Operational Response (SOR) [1]. Chemical incidents may involve chemical warfare agents (CWA), toxic industrial compounds (TICs), or other threat agents. To inform policy makers of best practices and optimised methods for decontamination, evidence is generated utilising a combination of ex vivo skin (in vitro), animal (in vivo), or human volunteer studies [2]. While in vitro and in vivo studies can provide valid decontamination data, controlled human trials can be used to specifically assess the effectiveness of decontamination procedures in a simulated chemical incident, leading to better understanding of the impact of human variability and operational practicality [3]. To assess the removal of chemicals from skin, or the penetration (and subsequent systemic availability) of chemicals in human volunteers, simulants that mimic the physicochemical properties of a harmful chemical are used [2,4,5]. In the context of human studies, a chemical simulant is defined as a compound that mimics relevant physicochemical properties of a more toxic agent without being toxic itself at the proposed applied dose. Naturally, simulants in other contexts can be toxic, such as 2-chloroethyl ethyl sulphide (half mustard, a simulant for sulphur mustard); however, for human studies, it is essential that simulants are of low human toxicity.

Specific information on simulants for TICs is unavailable, and decontamination studies have mostly used simulants of commonly researched CWAs including sulphur mustard (methyl salicylate) [4,6,7,8,9,10,11,12,13,14], soman (diethyl malonate) [15,16], and sarin (ethyl lactate) [11,12]. The most widely used simulant in decontamination studies is methyl salicylate; however, its high volatility makes it limited in use when investigating sequential decontamination interventions over an elongated time period [3]. Recently, two studies conducted on human hair and skin [13,14] employed a novel simulant, benzyl salicylate, to simulate more persistent agents such as VX or Novichoks. It is important to investigate simulants of lower volatility to ensure that when conducting human studies involving multiple decontamination interventions, there is not a false-positive decontamination efficacy caused by volatilisation of the simulant.

To date, all major published decontamination studies have utilised liquid simulants only, so evidence supporting strategies for the decontamination of powders from casualties is lacking. Due to the ever-increasing mass transport of agrochemicals [17] and the rise of the illicit use of potent opioid analgesics in solid form such as fentanyl and carfentanyl [18,19], methods for powder decontamination are of utmost importance. Powders can be just as physicochemically diverse as liquids in terms of water solubility, particle size, and partition into organic solvents. It is therefore important that optimisations to current decontamination methods consider the possibility that powders may be involved in an incident.

Decontamination interventions need to be applicable to chemicals with a wide range of physicochemical properties. Testing interventions on a range of physiochemically divergent simulants will ensure methods are suitable for use in incidents involving most chemical agents. This systematic literature review identifies chemicals previously applied to humans, screens them for suitability for application as simulants in human trials, and sorts them by physicochemical properties. The output is the creation of a repository of chemicals to facilitate selection of suitable simulants for future human decontamination (or related) studies.

## 2. Materials and Methods

### 2.1. Literature Search Strategy

Keywords used to build the search terms were derived from topics including human testing, skin absorption, and biomonitoring. Wildcard searches were applied to include a wide array of literature and were tailored for the syntax of each database.

For ease of translating syntaxes, the following databases were searched in order: Medline through the OVID Technologies Inc. search engine (Unrestricted date: 1946–July 2020, “in-process, in-data review, and other non-indexed citations”); SCOPUS as it covers a larger range of multidisciplinary journals than Medline; and finally, EMBASE and PubMed through the Healthcare Databases Advanced Search (HDAS) system.

Relevant papers in the reference sections of included studies were also screened. Bias was minimised through use of the PRISMA methodology for systematic reviews [20].

Full searches and results can be found in Table 1.

### 2.2. Inclusion Criteria and Literature Screening

Only articles written in English were included. The publication status was unrestricted (peer reviewed publications, grey literature etc.). Following the PRISMA methodology, firstly the titles of papers were screened for relevance. Those that met the criteria were subject to assessment of abstract, and any that the chemical could not be identified from were reviewed in full. The conforming articles made explicit reference to testing chemicals on humans, or they were discounted. Initially, only dermal studies were included; however, one compound (rosmarinic acid) was included based on a study of oral intake due to the large dose taken in the study and the low dermal toxicity of the compound.

### 2.3. Chemical Screening

To screen compounds for safety, the Globally Harmonized System of Classification and Labelling of Chemicals (GHS) statements were examined on the National Center for Biotechnology Information PubChem database [21] and the available online Material Safety Data Sheets (MSDS). A number of chemicals identified from the literature that have been studied using human volunteers in the past are no longer considered to be suitable for use in human volunteer trials. A colour coded system was applied to categorise chemicals; red for unsuitable, orange for “requires more data”, and green for potentially suitable. This system aimed to assess the risk of using the chemical as a simulant by assessing hazard alongside the assumption that simulants can be used in relatively large quantities (up to 1 mL) [22] and in an undiluted form to both simulate actual chemical incidents and to avoid any effects the diluent may have on skin penetration and/or decontamination efficacy.

Red category: Chemicals that are currently banned (either under the Stockholm Convention or through the European Union’s Registration, Evaluation, Authorisation, and Restriction of Chemicals (REACH) regulations) or classified as either carcinogenic, teratogenic, or mutagenic (or suspected) are acutely toxic, corrosive, chemical warfare agents from military trials, or are pharmaceutical products specifically tested on patients with health conditions. Acute toxicity (dermal, oral, and inhalational) was defined as any chemical with the GHS classifications H300—Fatal if swallowed, H301—Toxic if swallowed, H310—Fatal in contact with skin, H311—Toxic in contact with skin, H330—Fatal if inhaled, and H331—Toxic if inhaled. These chemicals were removed from further analysis. Chemicals that were removed because of “Dermal corrosion/irritation” were those that had the following GHS hazard statement: H314—Causes severe skin burns and eye damage.

Orange category: Chemicals with limited available data, GHS statements that included irritation and sensitisation but no further hazards, and chemicals where data sources had contradictory information. While these compounds may be used for the purpose of decontamination studies, they were not assessed in this review due to uncertainty over potential toxic effects and the requirement to conduct a comprehensive risk assessment dependent upon the doses to be utilised.

Green category: Chemicals classified as nonhazardous according to the European Chemicals Agency (ECHA), no GHS hazard statements (not including chemicals with no available data), or chemicals that had been previously used successfully in human decontamination trials (excluding any that automatically fall into the red category).

### 2.4. Physicochemical Property Identification

Physicochemical properties of chemicals in the green category were ascertained from scientific literature and online databases including PubChem, the ECHA registration dossier; MSDS; and peer reviewed literature that have adhered to regulated tests for physicochemical parameters (e.g., dermal toxicity measured by in vivo studies following OECD 402).

The physicochemical properties of interest and reasons for inclusion are in Table 2.

Some of the chemicals identified had specific structural isomers that are commonly misnamed within chemical searches (e.g., searches for 2-ethylhexyl salicylate (CAS #—118-60-5) regularly return information for octyl salicylate (CAS #—6969-49-9)). As a result, to identify the correct physicochemical properties, the Chemical Abstracts Service Registry Number (CAS) or the International Union of Pure and Applied Chemistry (IUPAC) name were used for all physicochemical searches. While chemicals of similar states have highly variable physicochemical properties, the largest differences were seen between solids and liquids; therefore, the results will be split by state at room temperature.

## 3. Results

**Figure 1:**
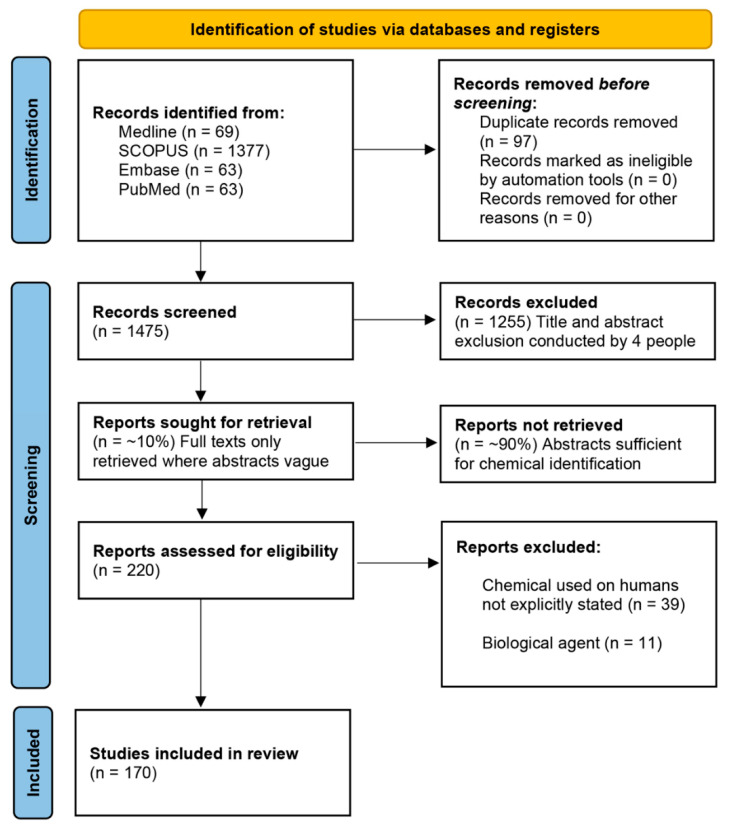
The PRISMA flow diagram showing the stages of screening and exclusion. From 1475 unique papers, 170 included chemicals that were assessed. This figure also shows the distribution of screened chemicals.

The systematic search of all databases yielded a total of 1572 results, reduced to 1475 following deduplication. Most papers did not mention the chemical in the title, so no papers were rejected based upon title alone. All 1475 publications were assessed according to their abstract for reference to the chemical(s) name and use on human volunteers. The PRISMA flow diagram is shown in Figure 1.

### 3.1. Chemical Screening

Of the 170 papers included for data extraction, a total of 171 unique chemicals were identified. A total of 78 chemicals were assigned to the red category, 54 to orange, and 39 to green (Table 3 and Table 4).

The red category includes the banned organochlorine pesticide aldrin, organophosphate pesticides azinphos-methyl and monochrotophos, and the toxic metal-containing compounds hexavalent chromium and mercuric chloride. Also included in this category were lower molecular weight compounds such as the solvents ethyl acetate, dichloromethane, and toluene for their high risk to respiratory pathways and, in some cases, acute single application organ toxicity in human.

Chemicals that were mild to moderately toxic or had limited data available were placed into the orange category. These denote chemicals that may be suitable for use as simulants on human volunteers, but either require more information on their toxicity to human, or potential caveats to their use. For example, skin sensitizers could be used, but only at concentrations below those that would initiate sensitization or could induce a reaction in sensitized individuals. Due to either a lack of available toxicological data or high uncertainty in identifying doses safe to use on humans, the chemicals in the orange category were not considered further. However, depending on the dosage used, the methodological design, and the context of the study, the authors do not rule out that with proper precautions, these chemicals may be used in studies involving human dermal exposure. The full list of red and orange category chemicals can be found in Table 3.

**Figure 2:**
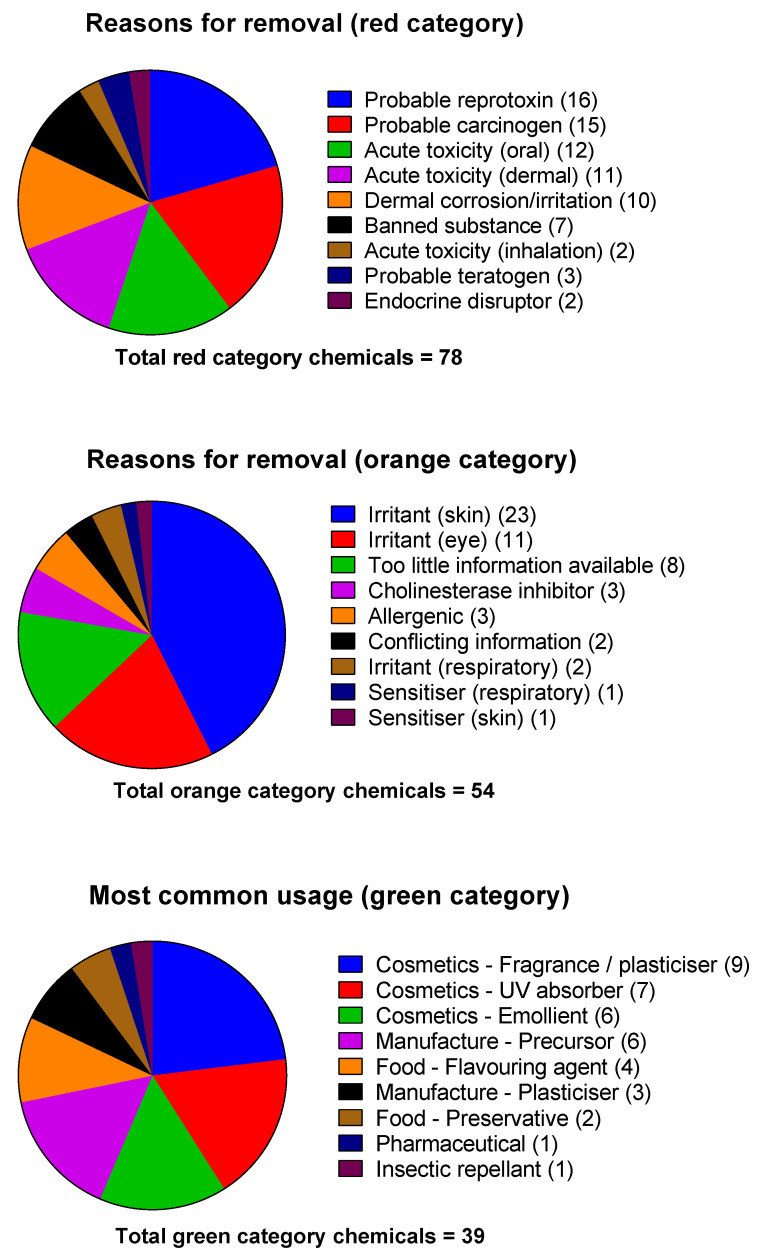
Exclusion characteristics of red and orange category chemicals, and the source/most-common commercial use of the green category chemicals.

Chemicals that fell into the green category included benzyl and methyl salicylate, which have been used in human volunteer decontamination studies [4,13,14], low toxicity insect repellent such as *N*,*N*-Diethyl-meta-toluamide (DEET), and plant secondary metabolites and derivatives such as ammonium glychryzzate, rosmarinic acid, and curcumin. Predictably, many chemicals found in commercially available topical sunscreens were also identified. These included avobenzone, octocrylene, and octyl methoxycinnamate. Figure 2 shows the reasons for removal of red and orange category chemicals and the predominant use of the green category chemicals.

### 3.2. Physicochemical Property Mapping

The physicochemical properties of the 40 chosen chemicals covered diverse ranges. For example, the solid compound rosmarinic acid possesses a vapour pressure ten orders of magnitude lower than the liquid methyl salicylate, the liquid diethylhexyl sebacate has a logK_ow_ of 10.08, while solid disodium sebacate has a logK_ow_ of −4.9.

**Figure 3:**
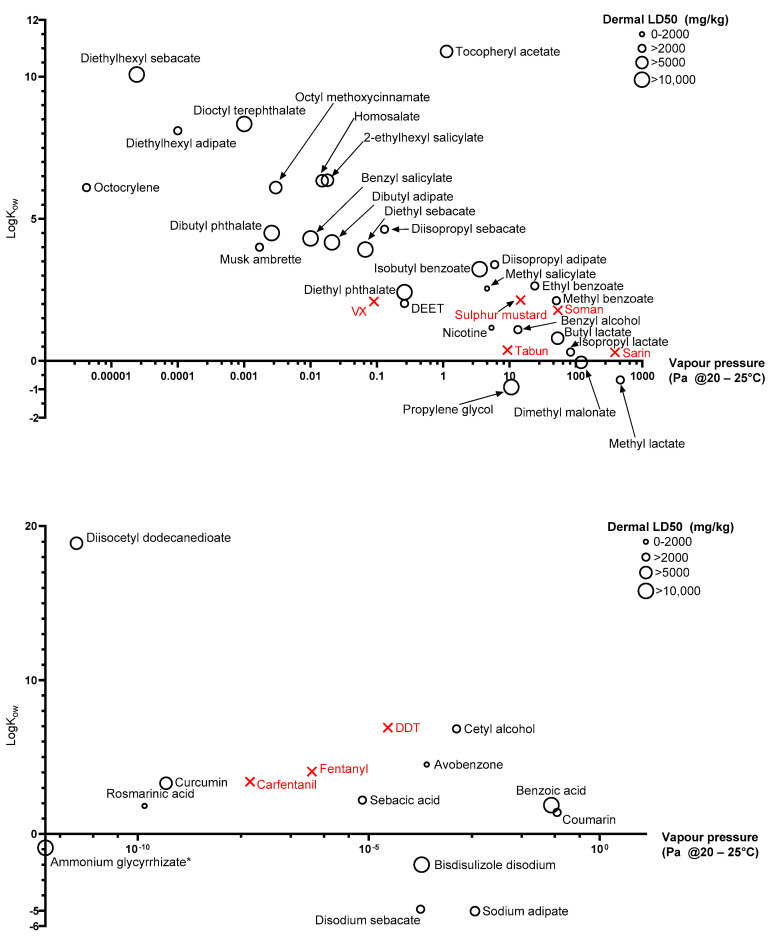
Physicochemical properties of room temperature liquids (top) and solids (bottom) in the green category. Ammonium glycyrrhizate (*) has 0 vapour pressure despite 0 being undefined on a logarithmic scale.

Due to varying magnitude of physicochemical properties identified, quantifying values as discreet values (low, medium, high) was not suitable. Discrete magnitude categories are subjective and what would be considered, e.g., high in this study, may be low in another based on the compounds of interest. Instead, key physicochemical properties are displayed in Figure 3 as scatter plots. Dermal LD50 values were for rat, rabbit, or guinea pig and, where data was available from more than one species, the lowest is presented. There were 7 chemicals that did not have available dermal toxicity data, so LD50 values were used from alternative routes of application: Ammonium glycyrrhizate (Oral—Mouse), diethyl sebacate (Oral—Rat), diisopropyl sebacate (Oral—Mouse), diisocetyl dodecanedioate (Oral—Rat), isopropyl lactate (Intramuscular—Guinea pig), methyl lactate (Oral—Rat), and rosmarinic acid (Intravenous—Mouse).

**Figure 4:**
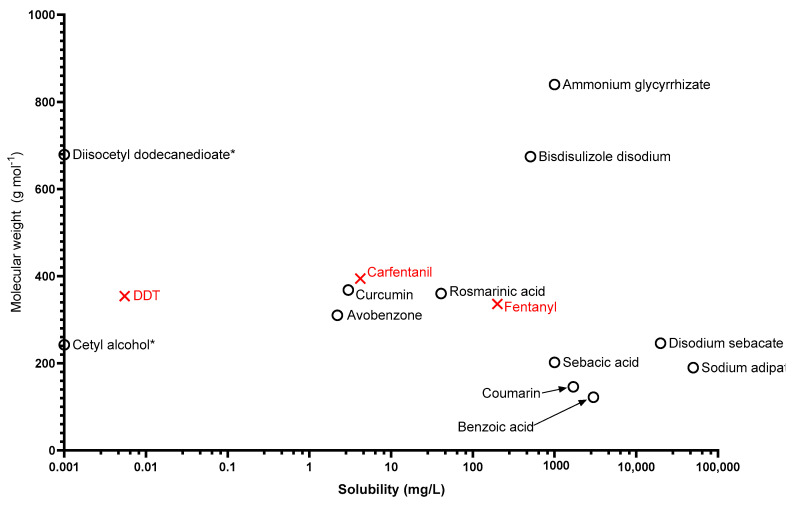
The solid compounds in the green category are plotted according to water solubility and molecular weight. (*) indicates 0 mg/L solubility despite 0 being undefined on a logarithmic scale.

In addition to logK_ow_ and vapour pressure, the water solubility and molecular weights of solid chemicals are represented in Figure 4.

The full physicochemical property list of green category chemicals can be found in Table 4.

## 4. Discussion

While this review has focussed specifically on retrieving data from studies in which chemicals have been applied to humans, it was inevitable that due to the unrestricted article publication date, that some studies would have utilised chemicals that would not be approved for use today. This is particularly the case for the series of studies conducted by Feldmann et al. [59] in which agrochemicals such as Aldrin, Monocrotophos and parathion were applied to the skin of volunteers. This study, published in 1974, reported the subsequent urinary excretion of carbon 14 radiolabelled versions of a range of pesticides also including azinphos-methyl (guthion) and diquat that were still in legal use, at the time this study was published. 

The most common reason for chemicals to be excluded from this study was their subsequent identification as carcinogens, mutagens, reprotoxins and/or teratogens. While each chemical was only labelled with one reason for exclusion, many chemicals reported a combination of toxicities such as trichloroethylene which is both carcinogenic and mutagenic. Where this occurred the first reason for removal found in the literature was chosen. Although exposure levels responsible for these detrimental effects are often far greater than would be utilised in decontamination studies, there is no reason to include such hazardous chemicals when their physicochemical properties are represented by less hazardous alternatives. 

Due to the nature of decontamination studies, dermal toxicity was seen as the most appropriate measure for toxicity in this review, however inhalational toxicity must be taken into account when utilising simulants with high volatility/vapour pressure. Oral toxicity is only relevant because during realistic decontamination protocols volunteers remove the contamination in indiscriminate ways, with potential for hand-to-mouth transfer or spread to more sensitive areas of the body such as the face, eyes or nose. While dermal studies are conducted to limit contact between the simulant and the mouth, it cannot be fully mitigated. Therefore, it is not advised that any chemical carrying the GHS classifications outlined in the red category are used as simulants as the risk of accidental intake cannot easily be controlled. 

The chemicals removed for dermal corrosion/irritation tended to be acids such as lactic acid, or alkalines such as sodium silicate. In addition, the metal containing complex mercuric chloride was excluded for reasons of corrosion, both to skin and mucosal membranes; however, the compound is also categorised as acutely toxic, mutagenic, and reprotoxic. 

Two chemicals were included in the red category for their intrinsic physiological properties. Estradiol and 5α-dihydrotestosterone are both hormones for reasons of ethical consideration and possible difficulty in identification of the exogenous contribution of the simulant during biomonitoring, use of these hormones are not considered further. 

While chemicals in the orange category have been excluded from consideration for use as simulants, it is important to note that some of the chemicals may be suitable for application to humans, if the dose applied and the risk assessments in place are appropriate. Benzophenone-3 is a common ingredient in UV-protective sunscreens and is licenced for use in formulations up to 6% *w*/*w* [60] due to the low risk it poses to human health but it is placed in the orange category due to the possibility of skin, eye, and respiratory irritation, and its contact and photo-allergenic potential, especially if applied undiluted. Simulants are most commonly applied undiluted as the diluents may enhance or retard penetration due to inter-simulant interactions or through changes to the conditions of the skin (e.g., the common penetration enhancer urea causes hydration of the stratum corneum [61]). As a result, undiluted benzophenone-3 would require a thorough risk assessment to be conducted to ensure the safety of the dose used in the study. Similarly, malathion has previously been used as a simulant for VX [62] and is the active ingredient in Derbac-M, a lice shampoo at a concentration of 0.5% *w*/*w*. Malathion is an organophosphate insecticide of relatively low dermal toxicity (dermal LD50—2330 mg/kg (rabbit)) which is readily metabolised into the more toxic malaoxon. In addition to the toxicity of its metabolites, the cholinergic effects of the required dose of malathion would need to be investigated prior to its use—for example, using the benchmark dose approach of the US EPA. Using this method, based on 20% inhibition of blood ACholE activity in rabbit at 127 mg/kg/d, with an uncertainty factor of 100, the benchmark dose would be 1.27 mg/kg/d [63].

Due to the toxicity of many of the chemicals in the orange category, any study would be heavily dose-dependent. For a controlled cross-over study design in which volunteers acts as their own control across multiple study sessions, multiple applications would be required on consecutive study sessions, increasing the potential for toxicity. Naturally, controlled cross-over studies should be designed in such a way that no residual dose is still present systemically or returned to baseline levels, however the possibility for “sub- chronic” accumulation cannot be negated including in target tissues. In addition, many compounds such as 2,4-D, ethyl paraben and malathion are sensitizers that could induce allergenicity when applied neat in multiple consecutive doses. 

logK_ow_ and vapour pressure are the most important physicochemical properties defining the usefulness of chemicals for assessment of dermal penetration, a commonly measured parameter in human decontamination studies. It is well known that lipophilic compounds (positive values of logK_ow_) have the ability to “pool” within the stratum corneum and deeper layers of tissue, a phenomenon sometimes referred to as the reservoir concept [64,65] and proportional to the magnitude of logK_ow_. Decontamination efficacy is chemical dependant, and logK_ow_ has a direct effect on this. 

A high vapour pressure indicates a compound’s “readiness” to evaporate or “off-gas” while a low vapour pressure indicates that a compound is persistent and unlikely to evaporate. In the context of environmental remediation Wyke et al. [66] previously quantified high vapour pressure as anything above 1.3 Pa and low vapour pressure below 1.3 × 10^−4^ Pa, with the range in between gradually increasing the likelihood of volatilising. Given the varying environmental conditions during a possible mass casualty incident however, these values should be used as nothing more than a rough guide. Vapour pressure is an important consideration during casualty decontamination. High vapour pressure contaminants may pose a respiratory threat but may also “self-decontaminate” through evaporation, while low vapour pressure compounds are less likely to pose a respiratory threat, but are more likely to persist and continue to penetrate skin and/or be transferred between casualties, first responders, and health personnel for a longer period of time. Vapour pressure is of particular importance to liquid simulants as solid simulants tend to be less volatile. The vapour pressures of the liquid simulants identified in this review range over eight orders of magnitude, with octocrylene being the most persistent liquid and methyl lactate being the least. The remaining liquid simulants identified are well dispersed through the range, covering each order of magnitude, ensuring that whether a high, medium or low vapour pressure simulant is required for a particular study, there are multiple simulant options available. The identified liquid simulants also cover a wide range of lipophilicities, with diethylhexyl sebacate being highly lipophilic (logK_ow_ = 10.08) and propylene glycol being relatively hydrophilic (logK_ow_ = −0.92). Figure 3 clearly also shows a trend between increasing vapour pressure and decreasing logK_ow_, as would be expected. 

Previous studies have focussed on using simulants specifically to represent one or two live CWAs, whereas recent studies [28,29] have focussed more on physicochemical diversity as being more important. If decontamination methods are effective against wider ranges of physicochemical properties, they are more likely to be effective against chemicals with wide ranging physicochemical properties, they are more likely to be effective when the contaminant identity is unknown. Despite this, it is clear that the simulants identified in this review overlap relatively well with physicochemical properties of agents of concern. Figure 3 (upper) shows five liquid threat agents, sulphur mustard, soman, tabun, sarin and VX, while Figure 3 (lower) and Figure 4 show three solid chemical threats, fentanyl, carfentanil and DDT. The compounds are relatively clustered towards the high vapour pressure, low logK_ow_ compounds; however, they still span almost four orders of magnitude in terms of vapour pressure and two orders of magnitude with regards to logK_ow_. Despite methyl salicylate being commonly used as a simulant for sulphur mustard, it is clear to see that the two compounds have fairly different vapour pressures and slightly different lipophilicities. It could be seen that the slightly more volatile ethyl benzoate would be a better simulant; however, this further highlights the limitations associated with choosing one simulant to represent only one agent of concern. Instead, the figure shows that a decontamination study conducted on multiple compounds of varying physicochemical characteristics would give the best understanding of the decontamination efficacy of interventions on unidentified chemicals in an emergency situation.

The Majority of published decontamination studies have focused upon removal of contaminants from skin [3,22,67] or hair [9,13,68,69]. Although this is extremely useful, especially considering transfer to first responders, other casualties, hospital staff, etc., it is less useful in interpreting likely improved outcomes for the exposed casualty. To understand this, a measure of systematic exposure such as blood or urine levels of simulants is more appropriate. When choosing simulants for a decontamination study that involves a measure of systemic exposure, the endogenous levels and exogenous sources of the proposed simulant should be taken into account together with how readily the simulant crosses the skin. 

The stratum corneum is an effective barrier against ingress of chemicals, and it is commonly accepted that compounds of mw >500 are less likely to penetrate this barrier without a carrier [70]. The solid simulants identified in this review range between 122.1 Da for benzoic acid and 840 Da for ammonium glycyrrhizate. While biomonitoring of simulants is becoming more widely accepted as an accurate measurement of decontamination efficacy [28], this relies on the simulant being able to penetrate the skin and become bioavailable. Of the 12 identified solid simulants, 9 are lower than 500 Da and could possibly be used for biomonitoring studies. However, the purpose of decontamination is not to just reduce the morbidity and mortality of the afflicted casualty, but to also reduce the likelihood of external contamination from spreading to unaffected casualties, first responders, or surfaces. In that regard, any of the solids identified in this review including the three above 500 Da could be used in an assessment of surface contamination, through tape stripping [71], simulant concentration in decontamination effluent, or simulant present on dry decontamination materials. 

For compounds that may be carried through the skin, the nature of the carrier can vary in composition. During wet decontamination, the potential carrier would be water or a soap–water solution which may carry chemical across the skin barrier, especially when the chemical is water soluble. It is worth noting that operational guidance and the recommended practices for the decontamination of powders by first responders varies between the UK and US. In the US, the Primary Response Incident Scene Management (PRISM) guidance [72] states that first responders should “use DRY decontamination unless contaminant is corrosive or in powder form”. This would imply that wet decontamination is recommended for the removal of solid contaminants in the US. UK recommendations, in contrast, states in respect to improvised wet decontamination that “water should only be used for decontamination where casualty signs and symptoms are consistent with exposure to caustic substances such as acids or alkalis or the contamination has been identified as biological or radiological in nature” [1]. Studies to identify the effect of water decontamination on solid contaminants would need to consider water solubility of the simulant. Comparisons could be drawn between highly water-soluble and water-insoluble powders, and effects related to hydration such as the wash-in effect [73] could be investigated. The powders identified through this review could be useful simulants as they cover a wide physicochemical range of water solubility (0–50,000 mg/L), vapour pressure (~0–0.12 Pa spread over 11 orders of magnitude), and partition coefficient (logK_ow_ range −5–18.9).

Factors not investigated in this study include the bioavailability and likelihood of penetration of the compounds and the feasibility of use of the chosen chemicals. High molecular weight compounds such as ammonium glycyrrhizate and diisocetyl dodecanedioate were included in this study as both were previously tested on humans; however, the studies they were involved in were Human Repeat Insult Patch Tests (HRIPT) evaluating skin irritation, rather than skin penetration. With molecular weights of 840 and 679.1 respectively it is unlikely that these compounds would be useful in dermal penetration studies, but they could be used for decontamination studies focused on removal from skin, hair, etc.

One limitation of this review is the lack of availability of LD50 data relating to humans or human skin surrogates (e.g., pig skin). The inclusion of such data would have given a better understanding of the likely toxicity of these compounds to humans; however, in the absence of such data, peer-reviewed and reliable LD50 values of other in vivo tests were included.

While this review recommends a particular list of chemicals for use as simulants based on their diverse physicochemical properties, it does not give any recommendations into the safe use of these chemicals, the dosages used in decontamination trials, nor the individual study design as decontamination studies can vary largely in objective and methodology. This review should therefore be seen as a tool to assist the choice of chemical simulant, which should be followed by rigorous risk assessment, ethical consideration, and study design.

## 5. Conclusions

Despite a wide array of chemicals being identified by this review, due to regulations, toxicities, and health hazards, the majority are not suitable for application to humans, especially in decontamination studies where volunteers routinely act as their own control over multiple conditions. A variety of chemicals were identified but not assessed due to specific requirements in risk assessment, dose control, and study methodology. The 40 chemicals identified that were deemed suitable to use (Table 4) varied greatly in lipophilicity and vapour pressure. Liquid and solid simulants represented a wide range of physicochemical properties and matched relatively well with the properties of threat agents. Whether investigating volatility, lipophilicity, solubility, liquid simulants, solid simulants, bioavailability, or surface contamination, this review has identified suitable chemical simulants that could be used in future decontamination studies.

## Figures and Tables

**Table 1:** Full systematic literature searches of the four databases. Wildcard searches are marked with an asterisk (*).

OVID—Medline (69 Results)		
No.	Search	Results
1	“Human volunt *”.tw.	9372
2	“Human test *”.tw.	3835
3	1 OR 2	13,183
4	Skin Absorption/	11,729
5	Skin penetra *.tw.	2250
6	Dermal penetra *.tw.	296
7	Percutaneous absor *.tw.	2109
8	Skin absor *.tw.	731
9	Dermal absor *.tw.	1002
10	Skin diffus *.tw.	130
11	Dermal diffus *.tw.	24
12	Skin applica *.tw.	546
13	Dermal applica *.tw.	712
14	4 OR 5 OR 6 OR 7 OR 8 OR 9 OR 10 OR 11 OR 12 OR 13	15,375
15	Urin *.tw.	496,262
16	Biomonit *.tw.	7693
17	Excret *.tw.	178,618
18	15 OR 16 OR 17	584,621
19	3 AND 14 AND 18	69
**SCOPUS (1377 Results)**		
(ALL (Human) W/1 ALL (Volunt * OR test *)) AND (ALL(skin OR dermal OR percutan *) W/2 ALL (penetra * OR absor * OR diffus * OR applica *)) AND (All(urin * OR biomonit * OR excret *))		
**HDAS—Embase (63 Results) and PubMed (63 Results)**		
(((Human ADJ1 (Volunt * OR test *)) AND ((skin OR dermal OR percutan *) ADJ2 (penetra * OR absor * OR diffus * OR applica *))) AND (urin * OR biomonit * OR excret *)).ti,ab		

**Table 2:** Physiochemical properties of interest for liquid and solid simulants, including the reason why that property is relevant to decontamination studies.

Physicochemical Property	Reason for Interest
Molecular weight	Molecules larger than 500 Da are unlikely to penetrate through skin
State (at room temperature)	Ability to separate liquid and solid simulants and to reject any gaseous chemicals
logK_ow_	Hydrophilicity/lipophilicity is a key parameter in skin and tissue penetration/sequestration and partitioning into water is key for wet decontamination
Vapour pressure (at 20/25 °C)	An indicator of persistence and possible inhalational risk
Dermal toxicity (LD50)	A measure of safety for human application; where not available, toxicity via other routes will be captured
Water solubility	To indicate the likelihood of dissolving in water during wet decontamination

**Table 3:** Red and orange category chemicals and their primary reason for exclusion.

**Reason for Removal (Red Category)**								
**Probable Reprotoxin**	**Probable Carcinogen**	**Acute Toxicity (Oral)**	**Acute Toxicity (Dermal)**	**Dermal Corrosion/Irritation**	**Banned Substance**	**Probable Teratogen**	**Acute Toxicity (Inhalation)**	**Endocrine Disruptor**
Beclomethasone dipropionate Budesonide Diflorasone diacetate Dimethylacetamide Dimethylformamide Ethylhexyl benzoate Fluazifop-butyl Methotrexate *N*-Methyl-2-pyrrolidone Penconazole Retinyl palmitate Styrene Tebuconazole Titanium dioxide Toluene Tris(2-ethylhexyl)trimellitate	1,1,1-trichloroethane 2,4,5-Trichlorophenoxyacetic acid Bromodichloromethane Carbaryl Chromium Dichloromethane Epoxiconazol Halometasone Hydroquinone Musk Ketone Musk Xylene Pirimicarb Propoxur Tetrachloroethene Trichloroethene	Cyfluthrin Deltamethrin Diclofenac sodium Ethion Flurbiprofen Indomethacin Ketoprofen Lindane Methyl formate Nonane Selenium Triclopyr	Cyanamide 1,3-dichloropropene (cis) Iloprost Laurocapram m-xylene *N*-Octyl bicycloheptene dicarboximide Nicotine p-phenylenediamine Promestriene Propetamphos Pyrethrin (unspecificed)	Ethyl glycolate Glycolic acid Lactic acid Lauryldimethylamine oxide Mercury (II) chloride *N*,*N*-Dimethylethylamine Sodium metasilicate Sodium silicate Stearamine oxide Tripropylene glycol diacrylate	Aldrin Dieldrin Diquat Guthion MGK 11 Monocrotophos Parathion	Dipyrithione Furosemide Trimethyl pentanyl diisobutyrate	3-Carene Methyldibromo glutaronitrile	Dihydrotestosterone Estradiol
**Reason for Removal (Orange Category)**								
**Irritant (Skin)**	**Irritant (Eye)**	**Not enough Available Information**		**May Cause Allergic Reaction**	**Cholinesterase Inhibitor**	**Conflicting Information**		**Irritant (Respiratory)**
2-butoxyethanol 2-methyl 1,3-propanediol 2-phenylphenol 4-aminobenzoic acid Borax Boric acid Butyl acetate Butyl benzoate Butyl paraben Capsaicin Captan Hydroxycitronellal Isopropyl alcohol Isostearyl alcohol Methyl glycolate Methyl tert-butyl ether Oxybenzone Permethrin Pyrene Pyroglutamic Acid Sodium lactate Stearyl benzoate Undecanedioic acid	Azelaic acid Benzaldehyde Butyl glycolate Dodecanedioic acid Dodecanol Ethyl acetate Ethyl butylacetylaminopropionate Ethyl lactate Methylparaben Salicylic acid Sodium benzoate	2-hexanone Dioctyldodecyl dodecanedioate Disodium octaborate tetrahydrate DMPS Isostearyl benzoate Piperonyl butoxide		2,4-D 2,4-D amine 2,4-D isooctyl ester Diisostearyl adipate Ethyl paraben Propranolol hydrochloride	Chlorpyrifos Diazinon Malathion	Enzacamene Glycyrrhizic acid MGK 326		Cypermethrin Propylene glycol methyl ether

**Table 4:** Full list of the chemicals in the green category and their physicochemical properties. The table is divided by state at room temperature.

	Chemical	CAS-Number	References	Molecular Weight	logK_ow_	Vapour Pressure (Pa at 20–25 °C)	Acute Toxicity	Solubility at 20/25 °C (mg/L)
**Liquids**	2-ethylhexyl salicylate	118-60-5	[23,24,25,26]	250.3	6.36	0.018	>5000 mg/kg—Dermal—Rat	<0.5
	Benzyl alcohol	100-51-6	[27]	108.1	1.1	13.3	2000 mg/kg—Dermal—Rabbit	40,000
	Benzyl salicylate	118-58-1	[28,29]	228.2	4.31	0.01	14,150 mg/kg—Dermal—Rabbit	8.8
	Butyl lactate	138-22-7	[30]	146.2	0.8	53	>5000 mg/kg—Dermal—Rabbit	40
	DEET	134-62-3	[31,32,33,34,35,36,37,38]	191.3	2.02	0.26	4280 mg/kg—Dermal—Rabbit	<1000
	Dibutyl adipate	105-99-7	[39]	258.4	4.17	0.021	19,000 mg/kg—Dermal—Rabbit	35
	Dibutyl phthalate	84-74-2	[40,41,42,43]	278.3	4.5	0.0026	20,000 mg/kg—Dermal—Rabbit	11.4
	Diethyl phthalate	84-66-2	[40,41,42,43,44]	222.2	2.42	0.26	>22,400 mg/kg—Dermal—Rat	932
	Diethyl sebacate	110-40-7	[39]	258.4	3.92	0.067	14,470 mg/kg—Oral—Rat	16
	Diethylhexyl adipate	103-23-1	[39,45]	370.6	8.1	0.0001	>2000 mg/kg—Dermal—Rat	0.0032
	Diethylhexyl sebacate	122-62-3	[39]	426.7	10.08	0.000024	>15,029 mg/kg—Dermal—Rabbit	<1
	Diisopropyl adipate	6938-94-9	[39]	230.3	3.39	5.946	>2000 mg/kg—Dermal—Rat	500
	Diisopropyl sebacate	7491-02-3	[39]	286.4	4.63	0.13	>2000 mg/kg—Oral—Mouse	2
	Dimethyl malonate	108-59-8	[39]	132.1	−0.05	120	>5000 mg/kg—Dermal—Rabbit	99,000
	Dioctyl terephthalate	4654-26-6	[44]	390.6	8.34	0.001	>19,680 mg/kg—Dermal—Guinea pig	0.0002
	Ethyl benzoate	93-89-0	[45]	150.2	2.64	24	>2000 mg/kg—Dermal—Rabbit	720
	Homosalate	118-56-9	[26]	262.3	6.34	0.015	>5000 mg/kg—Dermal—Rabbit	0.4
	Isobutyl benzoate	120-50-3	[45]	178.2	3.23	3.54	20,000 mg/kg—Dermal—Rabbit	98.3
	Isopropyl lactate	617-51-6	[30]	132.2	0.31	82.93	2500 mg/kg—Intramuscular—Guinea pig	183,000
	Methyl benzoate	93-58-3	[45]	136.2	2.12	50.66	>2000 mg/kg—Dermal—Rat	2100
	Methyl lactate	547-64-8	[30]	104.1	−0.67	466.6	>2000 mg/kg—Oral—Rat	Miscible
	Methyl salicylate	119-36-8	[11,12,14,22,46]	152.2	2.55	4.57	700 mg/kg—Dermal—Guinea Pig	625
	Musk ambrette	83-66-9	[47]	268.3	4	0.0017	>2000 mg/kg—Dermal—Rabbit	2.41
	Octocrylene	6197-30-4	[48]	361.5	6.1	0.0000042	>2000 mg/kg—Dermal—Rat	0.04
	Octyl methoxycinnamate	5466-77-3	[25,26,49,50]	290.4	6.1	0.003	>5000 mg/kg—Dermal—Rat	<1
	Propylene glycol	57-55-6	[51]	76.1	−0.92	10.6	20,800 mg/kg—Dermal—Rabbit	Miscible
	Tocopheryl acetate	58-95-7	[52]	472.7	10.89	1.12	>3000 mg/kg—Dermal—Rat	<1000
**Solids**	Ammonium glycyrrhizate	53956-04-0	[27]	840	−0.9	Presumed 0	12,700 mg/kg—Oral—Mouse	1000
	Avobenzone	70356-09-1	[50]	310.4	4.51	0.00018	>1000 mg/kg—Dermal—Rat	2.2
	Benzoic acid	65-85-0	[45,53]	122.1	1.87	0.09	10,000 mg/kg—Dermal—Rabbit	3000
	Bisdisulizole disodium	180898-37-7	[54]	674.6	−2	0.00014	>20 000 mg/kg—Dermal—Rat	509
	Cetyl alcohol	36653-82-4	[45]	242.4	6.83	0.0008	>2600 mg/kg—Dermal—Rabbit	0
	Coumarin	91-64-5	[55,56]	146.1	1.39	0.12	>2000 mg/kg—Dermal—Rat	1700
	Curcumin	458-37-7	[57]	368.4	3.29	4.1 × 10^−10^	>5000 mg/kg—Dermal—Rabbit	3
	Diisocetyl dodecanedioate	131252-83-0	[39]	679.1	18.9	4.7 × 10^−12^	>5000 mg/kg—Oral—Rat	0
	Disodium sebacate	17265-14-4	[39]	246.2	−4.9	0.000133322	>2000 mg/kg—Dermal—Rat	19,880
	Rosmarinic acid	20283-92-5	[58]	360.3	1.82	1.4 × 10^−10^	561 mg/kg—Intravenous—Mouse	41
	Sebacic acid	111-20-6	[39]	202.3	2.2	0.0000073	>2000 mg/kg—Dermal—Rat	1000
	Sodium adipate	7486-38-6	[39]	190.1	−5.03	0.002	>7940 mg/kg—Dermal—Rabbit	50,000

## Data Availability

All data is available upon reasonable request.
